# Early detection of left ventricular diastolic dysfunction using conventional and speckle tracking echocardiography in a large animal model of metabolic dysfunction

**DOI:** 10.1007/s10554-017-1287-8

**Published:** 2017-12-12

**Authors:** Mark M. P. van den Dorpel, Ilkka Heinonen, Sanne M. Snelder, Hendrik J. Vos, Oana Sorop, Ron T. van Domburg, Daphne Merkus, Dirk J. Duncker, Bas M. van Dalen

**Affiliations:** 1 Department of Cardiology, Thoraxcenter, Erasmus University Medical Center, ’s-Gravendijkwal 230, 3015 CE Rotterdam, The Netherlands; 2 Turku PET Centre, University of Turku, Turku, Finland; 3 Department of Clinical Physiology and Nuclear Medicine, University of Turku, Turku, Finland; 4 Department of Cardiology, Franciscus Gasthuis, Rotterdam, The Netherlands; 5 Division of Biomedical Engineering, Thoraxcenter, Erasmus University Medical Center, Rotterdam, The Netherlands

**Keywords:** Echocardiography, Speckle tracking echocardiography, Diastolic dysfunction, Left ventricular untwisting, Diabetes mellitus, Animal model

## Abstract

Left ventricular (LV) diastolic dysfunction is one of the important mechanisms responsible for symptoms in patients with heart failure. The aim of the current study was to identify parameters that may be used to detect early signs of LV diastolic dysfunction in diabetic pigs on a high fat diet, using conventional and speckle tracking echocardiography. The study population consisted of 16 healthy Göttingen minipigs and 18 minipigs with experimentally induced metabolic dysfunction. Echocardiography measurements were performed at baseline and 3-month follow-up. The ratio of peak early (E) and late filling velocity (E/A ratio) and the ratio of E and the velocity of the mitral annulus early diastolic wave (E/Em ratio) did not change significantly in both groups. Peak untwisting velocity decreased in the metabolic dysfunction group (− 30.1 ± 18.5 vs. − 23.4 ± 15.5 °/ms) but not in controls (− 38.1 ± 23.6 vs. − 42.2 ± 23.0 °/ms), being significantly different between the groups at the 3-month time point (p < 0.05). In conclusion, whereas E/A ratio and E/Em ratio did not change significantly after 3 months of metabolic dysfunction, peak untwisting velocity was significantly decreased. Hence, peak untwisting velocity may serve as an important marker to detect early changes of LV diastolic dysfunction.

## Introduction

Heart failure is a major public health problem in developed countries [1]. Left ventricular (LV) diastolic dysfunction is one of the important mechanisms responsible for symptoms in patients with heart failure, irrespective of the presence or severity of systolic LV dysfunction [2]. Diastolic dysfunction and filling pressures can be assessed by two-dimensional and Doppler echocardiography [3–5]. Speckle tracking echocardiography can be used to quantify several subtle changes in LV mechanics, for example LV twist and untwisting. As the base of the heart rotates clockwise along the LV long-axis, the apex rotates counter clockwise. This results into a wringing motion of the heart, defined as LV systolic twist and diastolic untwisting [6]. LV untwisting plays an important role in the mechanics of early LV filling [7].

Diabetes mellitus is an important risk factor for the development of LV diastolic dysfunction [8–11]. In previous studies on the use of speckle tracking for detection of LV dysfunction in diabetes mellitus, including two studies using a small animal model [12, 13], focus has been on assessment of LV strain and not on LV untwisting, and contrasting results were reported [14–23].

The aim of the current study was to identify parameters that may be used to detect early signs of LV diastolic dysfunction in a large animal model of metabolic dysfunction, using conventional and speckle tracking echocardiography.

Although it was shown in previous studies [24–26] that LV untwisting may be a meaningful parameter of diastolic function, its potential as an early marker of diastolic dysfunction is unclear. By longitudinally investigating a large animal exposed to important risk factors for development of diastolic dysfunction, our study may provide important added information in this regard. The hypothesis of our study was that LV untwisting may be useful for detecting LV diastolic dysfunction at a very early stage.

## Methods

### Porcine model of metabolic dysfunction

The study population consisted of 16 healthy Göttingen minipigs (mean age 17.8 ± 0.8 months, mean weight 31.1 ± 1.6 kg) and 18 minipigs with metabolic dysfunction (mean age 17.7 ± 0.6 months, mean weight 31.0 ± 1.3 kg). Diabetes was induced with intravenous (ear catheter) injections of streptozotocin (25 mg/kg/day) over 3 days (total dose 75 mg/kg). One week after diabetes induction, a high fat diet (25% of saturated fats and 1% of cholesterol) was gradually introduced for diabetic pigs, whereas control pigs continued on a normal chow diet [27]. Pigs were group-housed (a total of 9 pigs at the same time) with a separate individual ad libitum access to food for 1 h/meal, twice daily for the entire 3-month study duration. Studies were performed in accordance with the NIH Guide for the Care and Use of Laboratory Animals (8th edition, National Research Council. Washington, DC: The National Academies Press, 2011) and were approved by the Animal Care Committee at Erasmus University Medical Center Rotterdam.

### Echocardiography

**Fig. 1:**
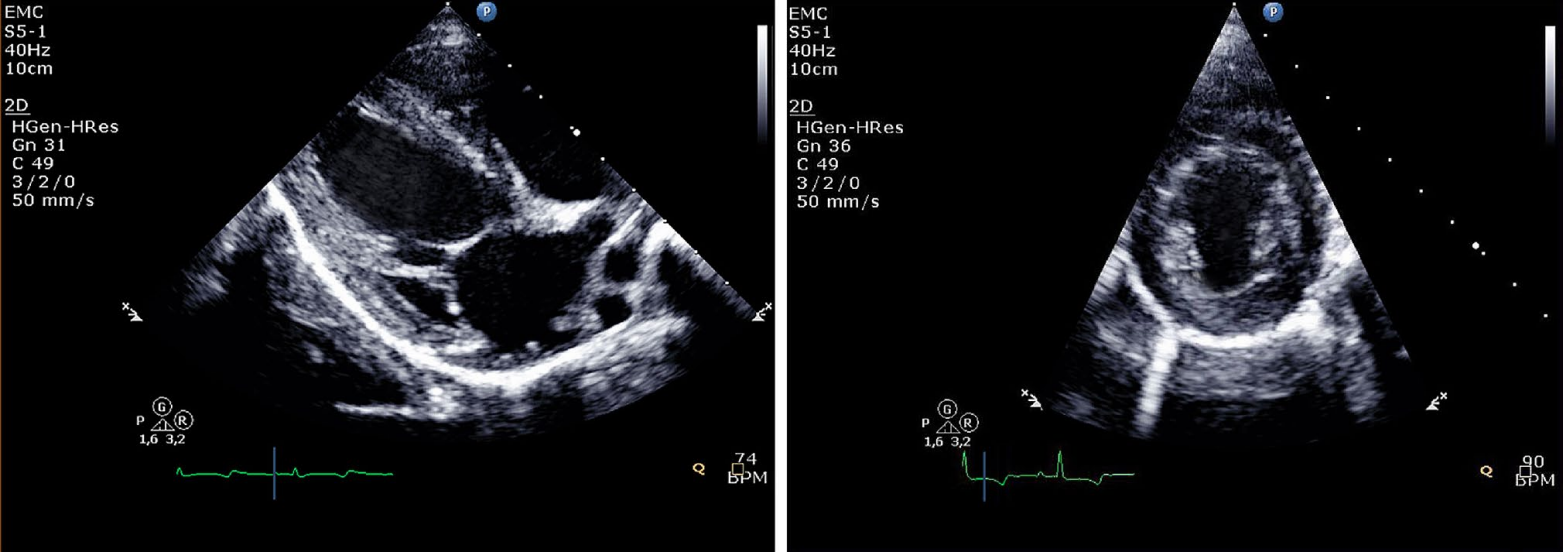
Example of a long-axis and short-axis echocardiographic image in a pig

Echocardiography measurements were performed at baseline and 3-months follow-up. Two-dimensional grayscale harmonic images were obtained in the right lateral decubitus position using a commercially available ultrasound system (iE33, Philips, Best, The Netherlands), equipped with a broadband (1–5 MHz) S5-1 transducer (frequency transmitted 1.7 MHz, received 3.4 MHz) (Fig. 1). Left atrial volume was calculated using the biplane area-length formula and indexed for body surface area [28]. From the mitral-inflow pattern, peak early (E) and late (A) filling velocities, E/A ratio, and E-velocity deceleration time (DT) were measured. Tissue Doppler imaging was applied by placing the sample volume at the side of the medial mitral annulus in an apical 4-chamber view [29]. Gain and filter settings were adjusted as needed to eliminate background noises and to allow for a clear tissue signal. To acquire the highest tissue velocities, the angle between the Doppler beam and the longitudinal motion of the investigated structure was adjusted to a minimal level. The velocity of the mitral annulus early diastolic wave (Em) was recorded at a sweep speed of 100 mm/s.

### Data analysis

Speckle tracking analysis of the datasets was performed using QLAB Advanced Quantification Software (version 10.0, Philips, Best, The Netherlands). In order to assess LV apical and basal rotation, tracking points were placed automatically and could be manually adjusted afterwards on an end-diastolic frame in each parasternal short-axis image, close to the endocardium. Rotation was defined as the mean angular displacement of all tracking points, relative to the centre of a circle through these tracking points. Clockwise rotation as viewed from the apex was expressed as a positive value, counter clockwise rotation was expressed as a negative value. LV twist was calculated as the difference between LV apical and basal rotation. Data were exported to a spread sheet program (Excel, Microsoft Corporation, Redmond, WA) to determine peak basal and apical rotation, peak twist, peak untwist velocity, and the timing of these parameters. Also, circumferential strain and strain rate (short-axis images) and longitudinal strain and strain rate (long-axis images) were measured.

### Statistical analysis

Statistical analyses were performed with SAS 9.3 program (SAS Institute, Cary, NC), using two-way ANOVA for repeated measures, with time (baseline vs. 3-month time point) and diabetes (controls vs. diabetic animals) as factors. If significant main effects or interactions were found, pairwise differences were identified with the Tukey–Kramer post hoc correction. A p value < 0.05 was considered statistically significant. Measurements were presented as mean ± SD. A Pearson’s R correlation test was conducted to examine whether there was a relationship between parameters that changed significantly from baseline to 3 months follow-up.

## Results

### Characteristics of the study population

Baseline characteristics are shown in Table 1. Diabetic pigs on a high fat diet developed significantly elevated glucose and lipid levels.

**Table 1:** General and blood characteristics of diabetic pigs on a high fat diet (n = 18) and healthy controls (n = 16)

	Baseline	3 months
Age at study onset (months)		
DM	17.7 ± 2.5	–
Control	17.8 ± 3.0	–
Body weight (kg)		
DM	31.0 ± 5.6	35.9 ± 7.3***
Control	31.1 ± 6.5	34.9 ± 5.4***
Glucose (mmol/l)		
DM	5.1 ± 1.0	12.9 ± 6.0^#^
Control	6.0 ± 1.1	5.5 ± 1.1
Triglycerides (mmol/l)		
DM	0.28 ± 0.09	0.94 ± 1.01^#^
Control	0.31 ± 0.08	0.29 ± 0.07
Total cholesterol (mmol/l)		
DM	0.94 ± 0.23	6.25 ± 4.51^#^
Control	0.93 ± 0.28	1.14 ± 0.27
LDL-C (mmol/l)		
DM	0.40 ± 0.11	3.76 ± 3.79^#^
Control	0.43 ± 0.18	0.44 ± 0.18
HDL-C (mmol/l)		
DM	0.55 ± 0.09	2.76 ± 1.24^#^
Control	0.52 ± 0.14	0.73 ± 0.16

Values represent mean ± standard deviation

*DM* diabetic pigs on a high fat diet

***p < 0.0001 as time effect; ^#^p < 0.01 as compared to controls at 3-months

### Conventional echocardiography

Baseline versus 3 months conventional echocardiography characteristics are shown in Table 2. E-wave velocity increased significantly after 3 months of metabolic dysfunction (52 ± 6 vs. 58 ± 10 cm/s) but not in controls (54 ± 9 vs. 53 ± 9 cm/s) whereas A-wave velocity increased significantly in both groups (43 ± 9 vs. 47 ± 11 cm/s in diabetic pigs on a high fat diet and 40 ± 7 vs. 46 ± 10 in controls). E/A ratio and E/Em ratio did not change significantly in both groups, although there was a trend towards an increase of E/Em ratio in the diabetic pigs on a high fat diet from 6.9 ± 1.5 at baseline to 7.7 ± 1.3 at 3 months (p = 0.06).

**Table 2:** Conventional echocardiographic characteristics of left ventricular diastolic function in diabetic pigs on a high fat diet (n = 18) and healthy controls (n = 16)

	Baseline	3 months
Left atrial volume (ml)		
DM	23 ± 6	25 ± 9
Control	26 ± 7	27 ± 8
Normalized left atrial volume (ml/kg)		
DM	0.77 ± 0.17	0.72 ± 0.29
Control	0.84 ± 0.20	0.79 ± 0.22
E-wave velocity (cm/s)		
DM	52 ± 6	58 ± 10^*^
Control	54 ± 9	53 ± 9
A-wave velocity (cm/s)		
DM	43 ± 9	47 ± 11^#^
Control	40 ± 7	46 ± 10^#^
E/A ratio		
DM	1.3 ± 0.3	1.3 ± 0.4
Control	1.4 ± 0.3	1.2 ± 0.2
E-wave velocity deceleration time (ms)		
DM	123 ± 30	126 ± 31
Control	118 ± 21	123 ± 28
Em septal (cm/s)		
DM	7.8 ± 1.4	7.7 ± 1.6
Control	7.7 ± 1.5	7.6 ± 1.5
E/Em ratio		
DM	6.9 ± 1.5	7.7 ± 1.3
Control	7.2 ± 1.4	7.2 ± 1.2

Values represent mean ± standard deviation

*DM* diabetic pigs on a high fat diet, *E-wave velocity* peak early phase filling velocity, *A-wave velocity* peak atrial phase filling velocity, *Em* peak early diastolic wave velocity

*p < 0.05 as compared to baseline, ^#^p < 0.05 as time effect

### Speckle tracking echocardiography

**Fig. 2:**
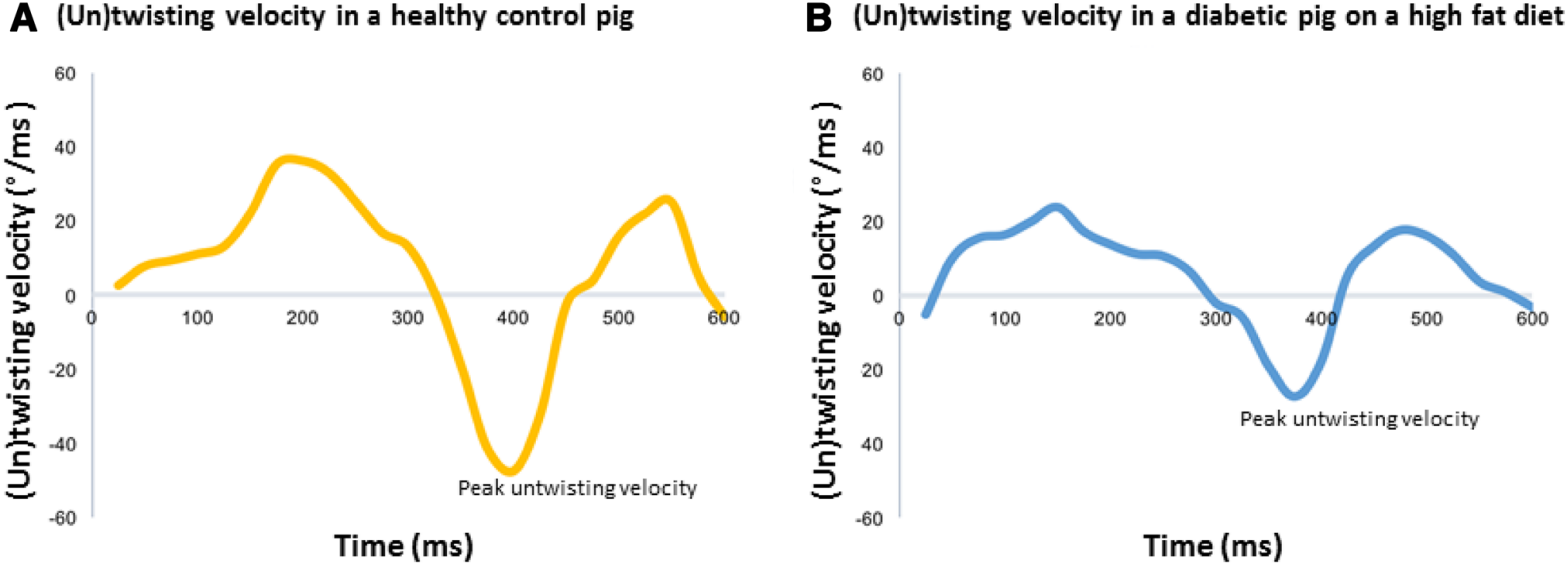
(Un)twisting velocity curves in a healthy control pig (**A**) and a diabetic pig on a high fat diet (**B**)

LV twist and untwist data are shown in Table 3. Most importantly, peak untwisting velocity (Fig. 2) decreased significantly after 3 months of metabolic dysfunction (− 30.1 ± 18.5 vs. − 23.4 ± 15.5 °/ms) but not in controls (− 38.1 ± 23.6 vs. − 42.2 ± 23.0 °/ms) resulting in a significant group difference at the 3-month time point (p < 0.05) while baseline values were not different (p = 0.698). No significant correlation was found between E or E/Em ratio and peak untwisting velocity (R^2^ = − 0.473, p = 0.111 and R^2^ = − 0.183, p = 0.481, respectively) or (delta) E or E/Em ratio and (delta) peak untwisting velocity (R^2^ = − 0.397, p = 0.121 and R^2^ = − 0.369, p = 0.145, respectively). LV longitudinal and circumferential strain and strain rate did not change significantly from baseline to 3 months in diabetic pigs on a high fat diet and healthy control pigs.

**Table 3:** Speckle tracking echocardiography parameters in diabetic pigs on a high fat diet (n = 18) and healthy controls (n = 16)

	Baseline	3 months
Systolic		
Peak rotation basal (°)		
DM	1.2 ± 1.2	0.8 ± 1.8
Control	0.7 ± 1.5	0.7 ± 1.5
Peak rotation apical (°)		
DM	4.4 ± 4.2	3.8 ± 4.2
Control	4.2 ± 2.6	4.1 ± 2.8
Peak velocity basal rotation (°/ms)		
DM	4.3 ± 3.0	8.8 ± 12.6
Control	5.5 ± 4.9	7.3 ± 5.6
Peak velocity apical rotation (°/ms)		
DM	− 26.6 ± 22.0	− 28.1 ± 32.7
Control	− 31.9 ± 29.7	− 21.1 ± 18.2
Peak twist (°)		
DM	4.1 ± 4.4	3.6 ± 4.4
Control	4.3 ± 3.8	3.7 ± 2.4
Time to peak twist (s)		
DM	0.4 ± 0.2	0.4 ± 0.1
Control	0.4 ± 0.1	0.4 ± 0.1
Diastolic		
Peak untwist velocity (°/ms)		
DM	− 30.1 ± 18.5	− 23.4 ± 15.5^*^
Control	− 38.1 ± 23.6	− 42.2 ± 23.0
Time to peak untwist velocity (s)		
DM	0.4 ± 0.1	0.4 ± 0.1
Control	0.4 ± 0.1	0.5 ± 0.1

Values represent mean ± standard deviation

*DM* diabetic pigs on a high fat diet

*p < 0.05 as compared to controls at 3-months

## Discussion

Peak untwisting velocity was significantly decreased in diabetic pigs on a high fat diet compared to healthy animals as assessed at 3-months from metabolic dysfunction onset. To the best of our knowledge, the current study is the first to show these early changes of LV diastolic dysfunction in a large animal model. These findings suggest that peak untwisting velocity may serve as an important marker to detect early changes of LV diastolic dysfunction.

Under normal physiological conditions, over 40% of diastolic LV untwisting has been completed after the first 15% of diastole, thereby contributing to the large and rapid pressure decay during the isovolumic relaxation phase [6]. This rapid, early LV untwisting process is the result of both active and passive mechanisms. There is a temporal dispersion in endocardial and epicardial repolarization, with in early diastole still depolarized endocardial fibres (as opposite to the already repolarized epicardial fibres) that may actively untwist the LV (normally the action of these fibres are overruled by the epicardial fibres) [7]. However, the effective force of contraction of myocardial fibres is expected to be minimal during this part of the cardiac cycle. Nevertheless, dissimilarities of apparent stiffness of the endocardium and epicardium caused by differences in detachment of actin-myosin cross-bridges may be of influence. Furthermore, high levels of stored potential energy from the active systolic twist are transformed into kinetic energy, adding a passive component to rapid early diastolic untwisting [30]. Subendocardial dysfunction in the diabetic pigs on a high fat diet may lead to loss of the active part of diastolic untwisting [10]. Also, increased stiffness of the LV may lead to a decreased potential to transform the potential energy stored in systolic twisting into rapid LV untwisting. Both of these phenomena may explain the decreased peak untwisting velocity found in the diabetic pigs on a high fat diet.

E-wave velocity increased significantly after 3 months of metabolic dysfunction and A-wave velocity increased in both groups. A possible explanation for this may be the increased circulating volume related to the increase in body weight over time in both groups, because the mitral inflow pattern is well-known to heavily depend on circulating volume status [4]. However, this leaves unexplained why E-wave velocity only changed in diabetic pigs on a high fat diet. It cannot be excluded that the latter does have to do with the development of LV diastolic dysfunction in these pigs. Nevertheless, in daily clinical practice qualification of LV diastolic function is based much more on E/A ratio than on the individual E-wave velocity or A-wave velocity values. Importantly, E/A ratio did not change significantly in both groups.

Although E/Em ratio increased by almost 12% after 3 months of metabolic dysfunction, this group difference failed to reach statistical significance. Increased E/Em ratio is essentially a marker of increased left atrial pressure. When LV diastolic dysfunction develops, LV filling will be impaired, subsequently leading to increased left atrial pressure [4]. Maybe the 3 month period was too short to allow development of significantly increased left atrial pressure. The results of our study show that at a time when peak untwisting velocity was already significantly decreased, E/Em ratio and E/A ratio failed to show significant differences between diabetic pigs on a high fat diet and controls.

After the 3 month period, both diabetic pigs on a high fat diet and control pigs were randomized to either a training program or an untrained, sedentary lifestyle. Unpublished data from our laboratory indicate that in this model at 5 months E/Em ratio was increased in untrained pigs with metabolic syndrome compared to untrained controls (8.6 ± 1.8 and 7.1 ± 0.9, respectively, p < 0.05). Therefore, development of diastolic dysfunction in the diabetic pigs on a high fat diet was confirmed by conventional echocardiography after 5 months.

In previous studies, abnormal values of LV strain, especially longitudinal systolic strain, in patients with diabetes mellitus have been reported [14–23]. Yet, in the current study we could not identify a decrease of longitudinal or circumferential systolic strain in the diabetic pigs on a high fat diet at a time when peak untwisting velocity was already significantly decreased. LV dysfunction is expected to start with diastolic dysfunction. Our finding therefore underscores the notion that the large animal model investigated in the current study may truly be a model of early LV dysfunction. Extension of the period of metabolic dysfunction may eventually lead to development of abnormal systolic LV strain values. Further studies are needed to test this hypothesis.

### Limitations

The study population is relatively small. Also, it is uncertain whether the findings in this large animal model may be extrapolated to humans. Therefore, the true potential of LV untwisting as an early marker of LV diastolic dysfunction should be confirmed in a prospective clinical study, for example in patients at high risk of development of LV diastolic dysfunction but with a normal echocardiogram according to conventional parameters.

## Conclusion

In this study, based on comprehensive conventional and speckle tracking echocardiography analyses of the LV in a large animal model of metabolic dysfunction, LV peak untwisting velocity was found to be a parameter for early detection of LV diastolic dysfunction.

## Abbreviations

LV: Left ventricle (or ventricular)
E: Peak early filling velocity
A: Peak late filling velocity
Em: Velocity of the mitral annulus early diastolic wave
